# Exploring Stroke Rehabilitation in Malaysia: Are Robots Better than Humans for Stroke Recuperation?

**DOI:** 10.21315/mjms2021.28.4.3

**Published:** 2021-08-26

**Authors:** Nik Nasihah Nik Ramli, Amhsavenii Asokan, Daniel Mayakrishnan, Hariharasudan Annamalai

**Affiliations:** International Medical School, Management & Science University, Shah Alam, Selangor, Malaysia

**Keywords:** robotic exoskeleton, physiotherapy, virtual reality, motor function recovery, stroke

## Abstract

Ranked as the second leading cause of death and the primary factor to adult disability worldwide, stroke has become a global epidemic problem and burden. As a developing country, Malaysia still faces challenges in providing ideal rehabilitation services to individuals with physical disabilities including stroke survivors. Conventional post-stroke care is often delivered in a team-based approach and involves several disciplines, such as physical therapy, occupational therapy, speech and language therapy, depending on the nature and severity of the deficits. Robots are potential tools for stroke rehabilitation as they can enhance existing conventional therapy by delivering a precise and consistent therapy of highly repetitive movements. In addition, robot-assisted physiotherapy could facilitate the effectiveness of unsupervised rehabilitation and thus, may reduce the cost and duration of therapist-assisted rehabilitation. Research on robot-assisted physiotherapy for stroke in Malaysia is slowly coming into the limelight in the past two decades. This review explores the effectiveness of robot-assisted physiotherapy particularly in improving motor functions of stroke survivors in Malaysia.

## Introduction

‘Stroke of God’s hand’ was the first lay term used for stroke since 1599, attributing to the sudden onset of symptoms to an acute event caused by a sudden interruption of blood supply to the brain (1–2). In 1970, the World Health Organization (WHO) defined stroke as a rapidly developing clinical sign of focal (or global) disturbance of cerebral function, with symptoms persisting 24 h or longer or leading to death (3). While this definition focuses on vascular origin etiology, the American Heart Association further included silent infarctions (inclusive of cerebral, spinal and retinal) and silent haemorrhages as observed though radiology (4). More recent terms, such as cerebrovascular accident (CVA), cerebrovascular insult (CVI) and brain attack, continue to be explored as the medical field progresses.

Stroke is a global health problem due to its reputation as the second most common cause of death while being the leading factor to adult disability worldwide (3). Approximately 87% of stroke deaths were from low-and middle-income nations (3). Similarly, in Southeast Asian region, the incidence and mortality rates due to stroke were found to be higher in low-income countries (5). In Malaysia, the stroke epidemic estimated by the Institute for Health Metrics and Evaluation (2017) conveyed that stroke represents the third leading cause of mortality in Malaysia. Preliminary data on the project ‘Monitoring Stroke Burden in Malaysia (2017)’ found that on average, 92 stroke admissions occur each day across all Malaysian healthcare facilities nationwide. Of these admissions, 40% of stroke afflicted patients were of younger aged groups (less than 60 years old). This debilitating phenomenon further challenged the Malaysian healthcare services with almost 32 deaths per day. Stroke survivors were often burdened with multiple morbidities, with estimates of almost 7 out of 10 stroke-afflicted survivors became dependents in activities of daily living (ADL). The cost of stroke care management accounted for 33,812 admissions in 2016 alone amounted to almost RM180 million (6).

Generally, the signs and symptoms present are sudden numbness or weakness in the face or limbs, especially on one side of the body, altered mental state, blurring of vision, loss of balance or trouble walking and severe headache (6). Based on etiology, stroke can be classified into ischaemic stroke and haemorrhagic stroke, while brief occurrences of neurological dysfunction resulting from focal cerebral ischaemia that are not associated with permanent cerebral infarction are classified as transient ischaemic stroke (7). The most recommended treatment for ischaemic stroke is intravenous injection of a recombinant tissue plasminogen activator (tPA), such as alteplase (activase) that is given within 3 h–4.5 h of the onset of stroke (8). As for haemorrhagic stroke, clinicians will approach neurosurgical options which consist of clamping the base of the aneurysm or embolising the blood vessels to secure the blood supply to the brain. (9). It is important to emphasise that the recommended window period for stroke intervention is within 6 h (10). A recent study has extended this period to 24 h in cases where the patient has salvageable brain tissue called ischaemic penumbra (11). With advances in acute treatment of stroke, more patients will have higher chances of surviving stroke with varying degrees of disability (12).

## Stroke Rehabilitation

### Conventional Stroke Rehabilitation

Alongside acute stroke treatment, rehabilitation is also an important aspect that must be taken into consideration in the continuum of stroke care. The primary objectives of stroke rehabilitation are to prevent deterioration and promote improvement of motor functions while achieving the highest possible level of independence (physically, psychologically, socially and financially) within the limits of the persistent stroke impairments (12). A structured algorithm for stroke care should consider early rehabilitation timing, a qualified rehabilitation team and suitable duration of rehabilitation. These have been distinguished as important elements in advancing better general outcomes for stroke patients (13). Generally, rehabilitation is often provided through a team-based approach and involves various disciplines, such as physical therapy, occupational therapy, speech and language therapy, depending on the nature and severity of the deficits. It may be provided at home with outpatient therapy, home health therapy, inpatient rehabilitation facility or skilled nursing facility placement (14).

In Malaysia, discharged stroke patients are provided with post-stroke care at specialist clinics that are based in state referral centres and secondary/district hospitals, or at outpatient primary care facilities (15). The latter can range from hospital-based outpatient units or public health centres or even private general practitioner clinics. Similar services are also provided by private healthcare service providers in which patients will self-finance with or without using health insurance schemes (15).

There are limited resources available regarding the guidelines for stroke rehabilitation in Malaysia which is probably due to uncoordinated services provided among healthcare centres. According to the official portal of the Malaysian Ministry of Health (MOH), the objectives of physiotherapy treatment for stroke recovery are: i) to accelerate the pace of recovery of the neuromuscular system like voluntary limb movement, balance, coordination and ability to walk; ii) to accelerate functional activities; iii) to ensure optimal mobility and independence and iv) to prevent complications such as pressure sores, foot drop, contracture and respiratory complications (16). A number of illustrated manuals provided by MOH include passive exercises to maintain joint range for both upper and lower limbs, correct positioning of stroke patients, as well as shifting and lifting of patients (16).

Malaysia as a developing country still faces challenges in providing optimum rehabilitation services to people with disabilities which includes stroke survivors. For instance, the delivery and timing of rehabilitation services to stroke survivors are determined by post-stroke duration or pre-decided maximum duration for service utilisation, and not based on individual functional needs and recovery as recommended in current stroke rehabilitation evidence-based guidelines of the National Stroke Foundation (15). Apart from that, rehabilitation services are normally discontinued after one-year post-stroke in most rehabilitation centres, often without transfer of care plan. Moreover, lack of designated stroke rehabilitation wards and shortage of trained rehabilitation professionals are also hampering the optimal rehabilitation service provided during the acute and recovery stages (15).

The National Stroke Clinical Practice Guideline of Malaysia does not address transfer of care and longer-term post-stroke care beyond tertiary care, which may pose more challenges for healthcare providers (17). Besides that, a study conducted in 2013 found that there was lack of awareness among physicians regarding the role of neurorehabilitation for stroke patients (18). This led to approximately one third of patients (31.1%) were not referred to any rehabilitation facility for further assessment or intervention (18).

Research on conventional stroke interventions and post-stroke outcomes is also very limited. One retrospective observational study was the first attempt to document outcomes of stroke patients managed in the community with follow-up in public primary care health centres in Peninsular Malaysia after being discharged from hospitals for the acute stroke episode (17). A total of 151 patients were recruited from 10 public primary healthcare centres for the study. The study highlighted the lack of coordination of post-stroke care beyond the acute stroke phase (17). Based on the limitations discussed previously, it is therefore important to include novel motivational interventions to encourage some level of non-clinician management for better results to overcome the limitations and challenges of conventional rehabilitation programmes.

## Robotic Rehabilitation for Stroke Recovery in Malaysia

Robotics is a field involving programmable multifunction devices that are designed to perform a task through various programmed motions (19). Medical robots were invented for multiple reasons, such as for surgical aid and rehabilitation aid (20). Rehabilitation robotics is a relatively young and emerging field that is currently being used in various countries.

Rehabilitation robotics is classified based on their functions and indications. There are assistive robots, prosthesis, orthotics and therapeutic robots. Assistive robots’ function is to improve one’s quality of life with severe or degenerative disabilities. It is helpful for patients with motor or cognitive limitations and can also be used to substitute a loss of function (21). Prosthesis is a mechanical device offered to patients with limb loss. It functions to provide mobility or manipulation abilities when a limb is lost or amputated. Orthotics, on the other hand, is a mechanism to assist or support a weakened joint, muscle or limb. These exoskeletons have links and joints similar to the human anatomy which assist the patient to move his/her limbs and to lift external loads. Therapy robots are commonly used in robotic rehabilitation centres and hospitals to increase and expand patients’ ability to regain motor function. In Malaysia, several rehabilitation centres are currently using different types of therapeutic robots which include upper limb therapeutic robots and lower limb therapeutic robots (Table 1).

Compact Rehabilitation Robot Haptic (CR2-Haptic) rehabilitation robot is designed for stroke patients with wrist and forearm weakness. It is portable and a cheaper alternative compared to other rehabilitation robots in Malaysia. The CR2-Haptic robot is a one-degree freedom robot that uses virtual reality games to improve one’s wrist and arm motor function. The robot can be used in three modes which are assistive mode, passive mode and active mode. Assistive mode functions to help the patient complete a task in the game (water drop collection). If the patient can complete the task within a certain period of time in assistive mode and passive mode, they would then be able to proceed to active mode. In active mode, the robot will exert various amounts of resistance to strengthen the arm and wrist muscles. Passive mode is focused more towards proprioception rehabilitation as it enhances the sensation of joint movement and thus promotes dynamic and stability of the joints (22). There is an exoskeleton concept of upper limb robotics known as ARMin/Armin 2 that is currently being used in several private hospitals in Malaysia. This device is equipped with force and position sensors that function to mimic the natural movement of shoulder and arm which can prevent joint degenerations (23).

Rehabilitation therapies in the lower limbs are equally important because one third of the patients with central nervous system lesions suffer from permanent walking disabilities and those who are able to move may experience an asymmetrical gait (24). Examples of lower limb robotic devices are treadmill gait trainers such as LOKOMAT and Lower Extremity Powered Exoskeleton (LOPES). The LOKOMAT consists of robotic orthosis with an advanced body weight distribution system that is combined with a treadmill. It uses computer-controlled motors which are integrated in gait orthosis located in the hip and knee joints (25). The function of these motors is to adjust the speed of the treadmill with the movement speed of the gait orthosis. On the other hand, LOPES is a lower limb therapeutic robot for gait training. It functions to improve the motion and activity of stroke patients and those with central nervous system impairments. The advantage of using this robot is that it helps the body and mind of a patient to recover naturally and is able to detect mistakes of patients while providing a suitable and correct realignment to the patient’s disabilities (25).

Robots are potential tools for rehabilitation as they can help to enhance existing conventional therapies. By using robots, therapies can be done in a precise and consistent manner especially for therapies that involve highly repetitive movements. Therapeutic robots could also function in collecting data in a quantitative method to predict the prognosis of a patient. In addition, robot assisted therapy could increase the efficiency of unsupervised therapy and reduce cost by decreasing the amount of one-to-one therapist cost for patients (26).

## Research on Robotic Rehabilitation for Stroke in Malaysia

### Virtual Reality

Research on robot-assisted physiotherapy for stroke in Malaysia is scarce. Among the limited number of studies, three randomised controlled trials testing robot-assisted physiotherapy that used virtual reality (VR) for improving upper limb function of stroke patients were selected for review (27–29). All three studies had a similar objective of investigating the extent of the role of VR in the improvement of physical functions. The studies by Ahmad et al. and Singh et al. (27, 28) investigated on the upper limb function using VR as an adjunct with the standard conventional physiotherapy. Another study by Singh et al. in 2013 tested the physical function of participants by substituting conventional physiotherapy with VR games.

Singh et al. (29) in 2013 substituted a portion of the time of standard physiotherapy with VR games, where the experimental group went through 30 min of VR games followed by 90 min of physiotherapy while the control group went through 120 min of standard physiotherapy. This study used a sample size of 28 participants who completed post-intervention assessments, where 15 participants were in an experimental group and 13 were in a control group. The findings were measured using the timed up and go test (score), 30-sec sit to stand test (seconds), timed 10-metre walk test (metres/sec), 6-min walk test (metres), overall balance score (score) and Barthel index score (score). All scores focused on assessing the abilities of the patients to perform daily living activities. The control and experimental group showed equal amounts of improvement or effectiveness reflected by their scores. However, there were no significant results observed between-groups effects of the outcome measurements (29).

In the year 2017, the same investigators carried out a more comprehensive randomised controlled trial to examine the impact of VR on upper limb function and psychological well-being of stroke patients (27). This research experimented on a sample size of 15 stroke patients in Pusat Latihan Perindustrian dan Pemulihan (Disability Services and Support Organisation). Analysis on the psychological well-being that applied the Depression Anxiety Stress Scale (DASS) showed a significant difference (*P* < 0.05) in the subscale of anxiety assessment, whereas the depression and stress subscales showed no significant differences between the VR groups and the control group. The decrease in anxiety among participants may have been due to the gratification of using VR during the session. The effectiveness of VR in improving upper limb performance of stroke patients was assessed by using the capability of upper extremities (CUE) questionnaire scores. The results, however, showed no significant improvements among the patients after VR intervention. On the contrary, the reaction time for upper limb performance of the patients was measured using paired *t*-tests and Wilcoxon signed-rank test which showed significant improvement after VR intervention (27).

In 2019, Ahmad et al. (28) similarly investigated the effectiveness and improvement of sensory functions of stroke survivors with VR as an adjuvant to standard physiotherapy for upper limb function and general health. This study was conducted using a bigger sample size of 36 participants in both experimental (*n* = 18) and control (*n* = 18) groups with a mean age of 57 and 63 years old, respectively. As compared to earlier studies, this study assessed the participants’ motor function using the Fugl-Meyer assessment for upper extremity (FMA-UE), which is the gold standard of motor functions assessment with a high degree of sensibility. Secondly, the Wolf motor function test was also conducted to ensure consistent and reliable results of the motor functions. In measuring the therapies’ effectiveness on general health, the intrinsic motivation involuntary scale, Lawton instrumental activities of daily living scale and stroke impact scale were used. The study eventually concluded that VR intervention on the experimental group had no difference in its effectiveness on general health and motor functions as compared to the control group (28).

It should be noted that all three studies faced similar limitations which included small sample size, restricted sample of participants aged 55 years old and above with decreased static balance performance, and short and limited duration of intervention. Despite the mixed results across the three studies, several significant results may imply that the integration of VR games as an adjunct to standard physiotherapy by substituting a part of the time with VR games can effectively maintain physical function outcomes and daily living activities among stroke patients as compared to standard physiotherapy alone.

### Interactive Rehabilitation and Assessment Tool (iRest)

A study on a rehabilitation robot, iRest, was conducted at Universiti Tun Hussein Onn and Universiti Teknologi Malaysia in 2017. With a sample size of four patients (one male and three females), an experimental pilot study was conducted to identify the important parameters to assess hand function of stroke patients (30). This study also quantified potential benefits of robotic assessment of iRest to predict the conventional assessment score, where it was carried out on patients being assessed through robotic assessment modules using the iRest robot. Patients were instructed to perform tasks such as Draw I, Draw Circle and Draw Diamond. These tasks were carried out for 30 min with 10 min for each module. The data received from the iRest robot was filtered by Savitzky-Golay (SG) smoothing filter and statistically analysed using MATLAB, while the patient’s motor functions were reassessed using motor assessment scale (MAS) at the end of the study. This experiment found a strong correlation between the kinematics variable for all robotic assessment and clinical MAS score for upper extremity. Based on this finding, it can be concluded that iRest may have the potential in predicting MAS score. One limitation faced by the researchers was the compensatory actions done by patients during the robotic assessments which damaged the iRest robot, thus potentially provided false results for the subsequent trials (30).

### CR2-Haptic Rehabilitation Robot

Similarly, in 2017, a research on upper limb motor functions of stroke patients was conducted in several public hospitals, National Stroke Association Malaysia (NASAM) and Universiti Teknologi Malaysia (UTM). The study aimed to evaluate the effectiveness of portable and reconfigurable wrist robots (22). A design of a modular and reconfigurable rehabilitation robot, CR2-Haptic, was proposed, whereby the cost and size were reduced through adoption of different therapeutic end effect for different training movements on a single robot. The study assessed the effectiveness of CR2-Haptic in wrist and forearm trainings on stroke patients through a randomised controlled trial with a sample size of seven patients. The patients were exposed to robotic therapy for 30 min (15 min for wrist and 15 min for forearm) and 1 h 30 min of conventional physiotherapy for a period of six weeks. The patients were assessed based on the Fugl-Meyer assessment scale for forearm and wrist (FMA-FW), MAS-hand score and modified Ashworth scale (MdAS) score for passive and active range of movement for both forearm pronation-supination (PS) and flexion-extension (FE). Post-training statistical results with CR2-Haptic showed significant improvement on the FMA-FW. A significant improvement in active range of motion (AROM) was detected in both forearm pronation-supination and wrist flexion-extension after the robotic training. Several limitations detected in this research were the patient’s group heterogeneity, small sample size, patient’s preparation prior to experiment and most importantly, the lack of control group to differentiate the exclusive improvement or benefit from robotic therapy alone. This study also had an absence of follow-ups with the patients, which could have provided sufficient data on the beneficial effects of robotic rehabilitation using CR2-Haptic (22). From these research, it was evident that rehabilitation robots such as CR2-Haptic and iRest contributed to significant improvement in rehabilitating upper limb motor function of arm and wrist as compared to VR intervention based on similar assessment tools used.

Based on the findings, it can be concluded that robotic rehabilitation can be equally effective as conventional physiotherapy. However, there is a lack of evidence that can support the superiority of robot-assisted physiotherapy as compared to conventional physiotherapy, particularly in Malaysia. Robotic physiotherapy cannot replace the role of conventional physiotherapy, but it can be used as an adjuvant for a better outcome for patients. It is considered effective as it can detect precise movements in therapy sessions while aiding the patients when needed. Robots can also deliver a high-dosage and high-intensity therapy composed of repetitive movements, thus can potentially produce a better recovery outcome (26). Additionally, physiotherapy assisted by robots may also promote over-ground ambulation with usage of exoskeleton which could reduce risk factors for secondary stroke like obesity. Joint contractures in the hips, knees and ankle joints are caused by prolonged immobility and can be prevented by application of robotic physiotherapy, thus preserving the stability of joints and preventing their degeneration (26). The application of exoskeleton can improve patients’ interaction with family and friends as they can ambulate independently without depending on others (31). Robotic physiotherapy can also improve patients’ mental health as it can reduce post-stroke anxiety, thus motivating patients to work for a better outcome in their therapy (32).

In contrast, cost of robot-assisted physiotherapy is considered a common reason why patients decline the therapy as it is generally considered expensive as compared to conventional therapy. The lack of portability is another issue for robotic physiotherapy as it requires a designated infrastructure and cannot be installed in patients’ homes (30). Robotic physiotherapy also requires a designated well-trained robotic physiotherapist which Malaysia still lacks (22). The usage of exoskeleton has been reported to cause pressure injury if it is applied for a long period of time which could add further complication on stroke patients (33). Furthermore, the limited study and literature on the benefits of robotic physiotherapy outcome in Malaysia need to be expanded further. A study also stated that most stroke patients were not familiar with the touch of robots compared to the touch of a human in their recovery process (34). Furthermore, the introduction of robotics in the health sector could also reduce job opportunities for medical personnel which include conventional physiotherapists (35).

## Conclusion

Various ongoing studies have proclaimed that the presentation of automated gadgets into the field of stroke recovery with numerous reports depicted the adequacy of robot-assisted treatment for improving motor and ambulatory capacity in patients with stroke. However, there are moral and methodological limitations that obstruct the plan of double-blinded, randomised and controlled investigations of robot-assisted treatment in patients with stroke. In addition, there are also limited thorough reviews of conducted robot-assisted physiotherapy. Meta-analysis of robot-assisted treatment is complicated to be carried out due to the heterogeneity of the mechanical devices that may not suit the diversity of participants’ qualities and study designs in the literature. In this manner, it is critical to consider opinions of healthcare professionals from multidisciplinary fields in order to make the best inferences in improving robotic rehabilitation therapy. This review attempts to understand the significant impacts of robotic devices in improving motor functions of stroke patients, particularly in Malaysia. Robotic therapy for stroke rehabilitation is in a dynamic phase of progress and has achieved outstanding advances. Continuous improvement of automated innovation may upgrade the adequacy and lessen the cost of such gadgets. Such advances will hoist robot-assisted treatment to a standard remedial methodology in stroke recovery. In conclusion, robot-assisted therapy as an adjunct is potentially beneficial in facilitating standard physiotherapy for stroke patients.

## Figures and Tables

**Table 1:** Centres for robotic stroke therapy in Malaysia

Name of centre	Types of robot	Focus of rehabilitation
Sunway Medical Centre	LOKOMAT Pro V5 HOCOMA	Improving over ground walking function and independency Improve range of motion of lower limbs Improve walking speed and endurance Improve strength and balance
Prince Court Medical Centre	LOKOMAT Pro V6 HOCOMA	Spinal cord injury Stroke traumatic brain injury Multiple sclerosis Parkinson’s disease Orthopedic locomotion problems
NASAM centres Petaling Jaya (HQ) Ampang Pulau Pinang Melaka Ipoh Johor Kuantan Sabah	CR2-Motion CR2-Haptic	Strokes Traumatic brain injuries Neurological disorders resulting in hand and arm impairment Muscle function
SOSCO Neuro-Robotics Rehabilitation & Cybernics Centre (PERKESO)	Hybrid Assistive Limb (HAL)^®^ Robot Suit	Lower limb training
University Malaya Medical Centre (UMMC)	Armeo^®^Spring	Upper limb rehabilitation Unilateral partial upper limb paresis Self-initiated movement therapy Simultaneous arm and hand therapy in a 3D workspace Motivating exercises Increased therapy efficiency
	Advanced Robotic Advanced Gait Rehab - GEO	Re-learning of walking on a plane coupling with Functional Electrical Stimulation (FES) and virtual worlds can be implemented
